# Aspiration thrombectomy prior to percutaneous coronary intervention in ST-elevation myocardial infarction: a systematic review and meta-analysis

**DOI:** 10.1186/s12872-016-0285-4

**Published:** 2016-06-02

**Authors:** Regina El Dib, Frederick Alan Spencer, Erica Aranha Suzumura, Huda Goma, Joey Kwong, Gordon Henry Guyatt, Per Olav Vandvik

**Affiliations:** Department of Anaesthesiology, Botucatu Medical School, Unesp – Univ Estadual Paulista, São Paulo, Brazil; McMaster Institute of Urology, McMaster University, Hamilton, Ontario Canada; Division of Cardiology, Department of Medicine, McMaster University, St. Joseph’s Healthcare - 50 Charlton Avenue East, Hamilton, Ontario Canada; Research Institute - Hospital do Coração (HCor), São Paulo, Brazil; Department of Pharmacy, Tanta Chest Hospital, Tanta, Egypt; Division of Cardiology and Heart Education And Research Training (HEART) Centre, Department of Medicine and Therapeutics, Prince of Wales Hospital, and Institute of Vascular Medicine, The Chinese University of Hong Kong, Shatin, Hong Kong; Department of Clinical Epidemiology and Biostatistics, McMaster University, Hamilton, Ontario Canada; Department of Medicine, McMaster University, Hamilton, Ontario Canada; Department of Medicine, Innlandet Hospital Trust-Division Gjøvik, Oppland, Norway; Institute for Health and Society, Faculty of Medicine, University of Oslo, Oslo, Norway

**Keywords:** Myocardial infarction, Aspiration thrombectomy, GRADE, Systematic review, Meta-analysis

## Abstract

**Background:**

Trials of aspiration thrombectomy (AT) prior to primary percutaneous intervention (PCI) in patients with ST-segment elevation MI (STEMI) have shown apparently inconsistent results and therefore generated uncertainty and controversy. To summarize the effects of AT prior to PCI versus conventional PCI in STEMI patients.

**Methods:**

Searches of MEDLINE, EMBASE and CENTRAL to June 2015 and review of reference lists of previous reviews. We included randomized controlled trials (RCTs) comparing AT prior to PCI with conventional PCI alone. Pairs of reviewers independently screened eligible articles; extracted data; and assessed risk of bias. We used the GRADE approach to rate overall certainty of the evidence.

**Results:**

Among 73 potential articles identified, 20 trials including 21,660 patients were eligible; data were complete for 20,866 patients. Moderate-certainty evidence suggested a non statistically significant decrease in overall mortality (risk ratio (RR) 0.89, 95 % confidence interval, 0.78 to 1.01, risk difference (RD) 4/1,000 over 6 months), no impact on recurrent MI (RR 0.94, 95 % CI, 0.79 to 1.12) or major bleeding (RR 1.02, 95 % CI, 0.78 to 1.35), and an increase in stroke (RR 1.56, 95 % CI, 1.09 to 2.24, RD 3/1,000 over 6 months).

**Conclusions:**

Moderate certainty evidence suggests aspiration thrombectomy is associated with a possible small decrease in mortality (4 less deaths/1000 over 6 months) and a small increase in stroke (3 more strokes/1000 over 6 months). Because absolute effects are very small and closely balanced, thrombectomy prior to primary PCI should not be used as a routine strategy.

## Background

In patients with ST-segment elevation myocardial infarction (STEMI), primary percutaneous coronary intervention (PCI) rapidly restores myocardial flow resulting in decreased infarct size and decreased mortality compared to thrombolysis or conservative medical management [1]. Some patients may, however, experience distal embolization of thrombus and plaque debris with failure to adequately restore distal microcirculatory flow. This “no reflow” phenomenon is associated with an increase in infarct size and lower survival [2].

Randomized clinical trials (RCTs) comparing aspiration or mechanical thrombectomy prior to primary PCI to PCI alone have shown improvement in markers of myocardial reperfusion (e.g. “myocardial blush”, ST-segment resolution post procedure) [3]. A recent meta-analysis of 20 RCTs addressing patient-important outcomes and including over 11,000 patients reported that aspiration thrombectomy prior to primary PCI was associated with a reduction in major coronary adverse events and 1-year mortality [4]. A more recent meta-analysis including 26 RCTs, reported a different conclusion: aspiration thrombectomy did not improve clinical outcomes [5]. Neither of these meta-analyses included the recently published Trial of Routine Aspiration Thrombectomy with PCI versus PCI Alone in Patients with STEMI (TOTAL), which randomized over 10,000 patients [6].

We therefore undertook a systematic review of all RCTs comparing aspiration thrombectomy prior to PCI *versus* PCI alone in patients with STEMI, focusing on patient-important outcomes. As composite endpoints varied between trials and can produce misleading results [7, 8], we focused on individual endpoints of overall mortality, recurrent MI, stroke, and major bleeding.

## Methods

This review adheres to the Preferred Reporting Items for Systematic Reviews and Meta-analyses (PRISMA) Statement [9]; the Quality of Reporting of Meta-analyses QUOROM [10]; and the Cochrane Handbook for Systematic Reviews of Interventions [11].

### Eligibility criteria

We included RCTs that compared aspiration thrombectomy prior to PCI with conventional PCI in patients with STEMI, included any one of the following patient-important outcomes: overall mortality, cardiovascular (CV) mortality, myocardial infarction (MI), stroke (including ischemic and hemorrhagic stroke) and, non-fatal extracranial major bleeding, and followed patients for at least 30 days. We excluded studies reported only as conference abstracts.

### Data source and searches

A previous review with similar inclusion criteria identified studies up to December 2013 [5]. Using Medical Subject Headings (MeSH) based on the terms “thrombectomy,” “thrombus aspiration,” “thromboaspiration,” “infarction,” and “myocardial infarction” (Appendix Table 1) we replicated the search strategy of that review [5] for Medline, EMBASE, and Cochrane Controlled Trials Register (CENTRAL) from January 1, 2014 to June 26, 2015. We also reviewed reference lists of relevant review articles [4, 5, 12] and primary studies.

### Selection of studies

Teams of two reviewers independently screened all titles and abstracts identified by the literature search, obtained full-text articles of all potentially eligible studies, and evaluated these studies for eligibility criteria.

### Data extraction and risk of bias assessment

Three pairs of reviewers independently extracted the following data using a pre-standardized data extraction form: characteristics of the study design; participants; interventions; outcomes event rates and follow-up.

Reviewers independently assessed risk of bias by using a modified version of the Cochrane Collaboration’s tool for assessing risk for bias tool [13] (http:/distillercer.com/resources/) [14] that includes nine domains: adequacy of sequence generation, allocation sequence concealment, blinding of participants and caregivers, blinding of data collectors, blinding for outcome assessors, blinding of data analysts, incomplete outcome data, selective outcome reporting, and the presence of other potential sources of bias not accounted for in the previously cited domains [14]. For incomplete outcome data we stipulated as low risk of bias loss to follow-up of less than 10 % and a difference of less than 5 % in missing data in intervention and control groups.

### Certainty of evidence

The reviewers used the Grading of Recommendations Assessment, Development and Evaluation (GRADE) methodology to rate certainty of the evidence for each outcome as high, moderate, low, or very low [15]. Detailed GRADE guidance was used to assess overall risk of bias [16], imprecision [17], inconsistency [18], indirectness [19] and publication bias [20], and summarized results in an evidence profile. We assessed publication bias through visual inspection of funnel plots for 10 or more studies.

For decisions regarding eligibility, risk of bias assessment, and data abstraction, reviewers resolved disagreement through discussion with third party adjudication if necessary.

### Data synthesis and statistical analysis

We chose six months as a follow-up time that represented duration important to patients, sufficient to include most events that would likely be influenced by thrombectomy, and would include relatively few events that would not be potentially influenced by thrombectomy. For meta-analyses we used six months data if available; and otherwise we chose the time point closest to six months, but preferring 1-year over 30 days.

We calculated pooled risk ratios (RRs) and associated 95 % confidential intervals (CIs) using random-effects models with statistical method of Mantel-Haenszel. Absolute effects and 95 % CI were calculated by multiplying pooled RRs and 95 % CI by baseline risk estimates derived from the TOTAL study (the most recent and largest of the included RCTs) [6]. We addressed variability in results across studies by using I^2^ statistic and the P value obtained from the Cochran chi square test. Our primary analyses were based on eligible patients who had reported outcomes for each study (complete case analysis). For overall mortality we used all-cause mortality when available. For studies that did not present all-cause mortality we used cardiovascular mortality. We assessed publication bias through visual inspection of funnel plots for outcomes addressed in 10 or more studies. Review Manager (RevMan) provided the software for all analyses (version 5.3; Nordic Cochrane Centre, Cochrane) [21].

We also performed a meta-regression with a fixed-effect model using restricted estimated maximum likelihood with an observed log-odds ratio to predict whether mortality and recurrent myocardial infarction rates changed significantly by mean age. Meta-regression analysis was performed using Stata-13 (StataCorp LP, College Station, TX).

## Results

### Selection of titles

**Fig. 1:**
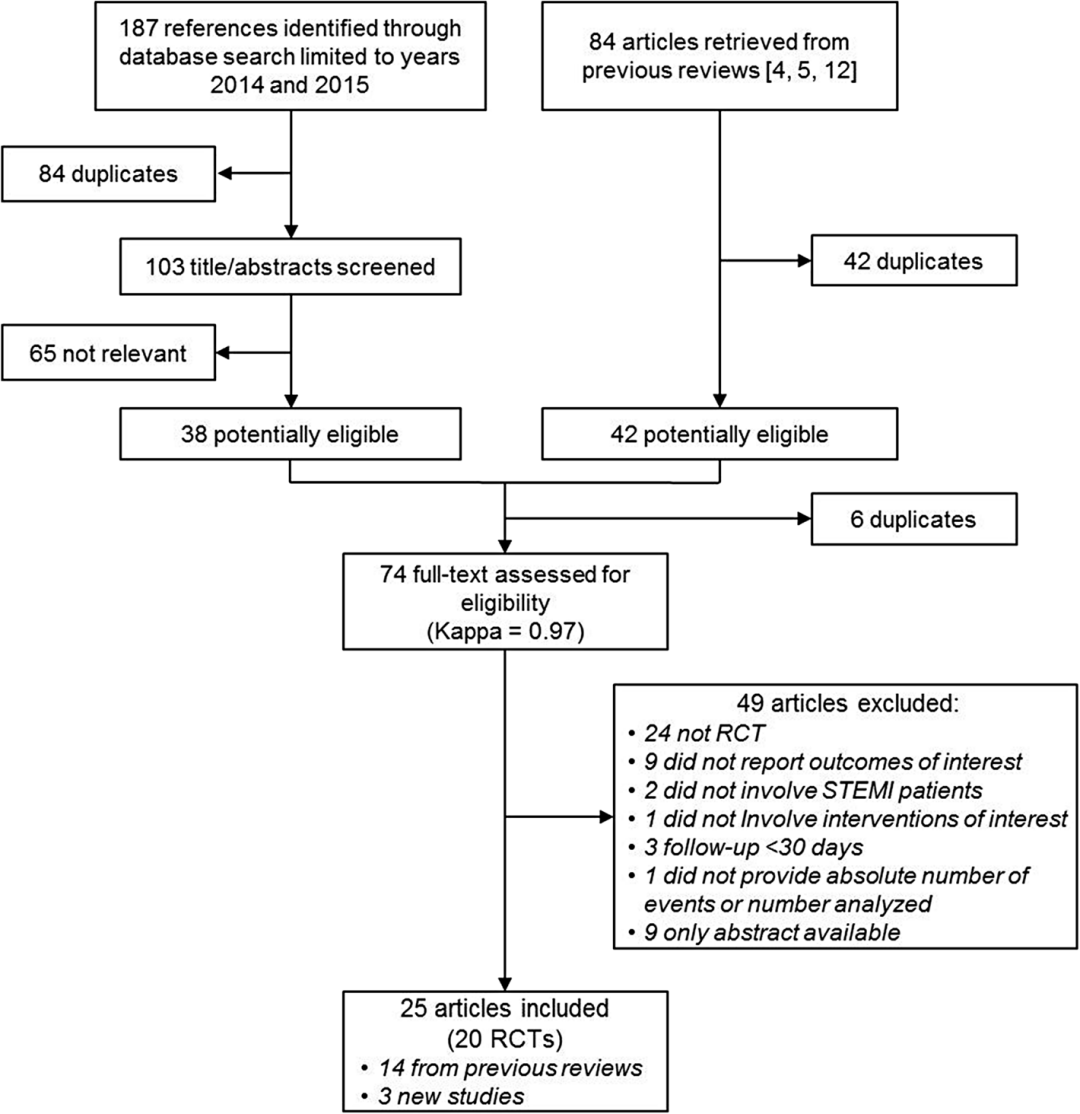
Flowchart of the review

Our search strategy focusing on publications since the last review identified 103 unique citations (Fig. 1). After title and abstract screening, we assessed the full-text version of 38 relevant citations. In addition, we identified 42 potentially eligible publications included in previous systematic reviews, six [6, 22–26] of which were also identified in our search strategy. Thereafter, we assessed eligibility of 74 unique publications and excluded 49 studies (Fig. 1). As a result, we included 25 publications documenting 20 randomized controlled trials [6, 25–48] involving 21,660 participants. Two studies [28, 35] and one updated follow-up [46] were not included in any of the previous reviews.

### Study characteristics

Ten studies [26, 27, 29, 31–34, 39–41, 43–46] were conducted largely in Europe (Table 1). Sample size ranged from 56 [35] to 10,732 [6] patients of whom a majority were males with mean ages typically in the early 60s. Studies included adult STEMI patients typically with symptoms lasting >30 min but <12 hours, and cumulative ST-segment elevation of >0.1 mV in ≥2 leads. Some studies excluded life expectancy < 6 months [6, 28, 29]; cardiogenic shock [28, 29, 32, 33, 35–38, 45–47]; previous CABG or MI or significant left main coronary lesion [6, 25, 29–33, 35–37, 39, 40, 42, 45–47]; pre-catheterization therapy with lytic agents [34]; severe asthma or bradycardia precluding use of adenosine [35]; dialysis; platelet count <100,000 or >700,000 cells/mm3; hemoglobin <10 g/dL [36, 37]; severe HF treated with intra-aortic balloon pump [39]; contraindication or prior use of platelet glycoprotein IIb/IIIa inhibitors [32–34, 40, 42]; rescue or facilitated PCI [42–44]; need for emergency CABG [26, 27]; pregnancy [45, 46]; and major planned surgery requiring discontinuation of antiplatelets agents [45, 46]. Follow-up time ranged from 30 to 360 days.

**Table 1:** Study characteristics

Author, year	Location	No. patient	Mean age (SD)	No. male (%)	Inclusion criteria	Exclusion criteria	Follow-up time (months)	Outcomes evaluated
ADMIT [28]	Haifa, Israel	100	I = 57.5 (12.4)	86 (86.0)	Admission <12 hours of onset of symptoms of STEMI, regardless of the initial TIMI flow	Inability to consent; known allergy to either aspirin or clopidogrel; life expectancy <6 months; cardiogenic shock	6 months	Quality of epicardial and microcirculation perfusion; LV function; ischemic mitral regurgitation; MACE (death, recurrent MI, TVR)
			C = 57.2 (12.1)					
Bulum 2012 [29]	Zagreb, Croatia	60	I = 54.3 (9.7)	47 (78.3)	Symptoms suggesting acute myocardial ischemia of >20 min, time from symptom onset of <12 hours, and ST-segment elevation >0.1 mV in >2 contiguous ECG leads	Need for rescue PCI after failed thrombolysis; cardiogenic shock; triple-vessel disease; significant LMCA stenosis; previous PCI of an IRA; previous CABG; life expectancy <6 months	6 months	Referent vessel diameter; minimal lumen diameter; lesion length; percentage of diameter stenosis; MACE (death, recurrent MI, stroke, TLR)
			C = 58.5 (8.6)					
Chao 2008 [30]	Taipei City, Taiwan	74	I = 60 (13)	63 (85.1)	STEMI (typical chest pain >30 min with new ST-segment elevation ≥0.1 mV in >2 contiguous leads on a 12-lead ECG), <12 hours after onset, and eligible for primary PCI	Killip IV hemodynamic status; ventricular tachyarrhythmias; previous CABG or significant LMCA lesion; culprit vessel diameter <2 mm; existing TIMI 3 flow without visible thrombus in IRA	6 months	Angiographic differences in TIMI and MBG (post PCI - baseline); MACE (death, stroke, non-fatal recurrent MI, TVR)
			C = 62 (11)					
De Luca 2006 [31]	Rome, Italy	76	I = 66.7 (14.1)	48 (63.2)	Anterior STEMI, >18 years old, and have an identifiable thrombus on IRA at coronary angiography	Previous MI or CABG; triple-vessel disease; severe valvar disease; TIMI 2 or 3 flow at the time of initial angiography; unsuccessful PCI defined as no antegrade flow or >50 % residual stenosis in the IRA	6 months	LV remodeling; MACE (death, recurrent MI, hospitalization for HF)
			C = 64.6 (12.5)					
EXPIRA [32, 33]	Rome, Italy	175	I = 66.7 (14.1)	105 (60.0)	First STEMI, <9 hours from symptoms onset, IRA ≥2.5 mm in diameter, thrombus score ≥ 3, TIMI flow ≤1, and >18 years old	Previous PCI on IRA; previous CABG; cardiogenic shock; triple-vessel disease; LMCA disease; severe valvular disease; thrombolysis; contraindication to glycoprotein IIb/IIIa inhibitors	9 months	Final MBG ≥2; rate of 90-min ST-segment resolution >70 %; MACE (cardiac death, recurrent MI, TVR); stent thrombosis
			C = 64.6 (12.5)					
EXPORT [34]	24 centres in India and Europe	249	I = 59.2 (12.8)	202 (81.1)	>18 years old, STEMI <12 hours of symptom onset, ST-segment elevation ≥2 mm in ≥2 contiguous leads, visual reference vessel diameter >2.5 mm, and with TIMI flow of 0 or 1 before placing the wire in the IRA	Cardiogenic shock; cardiac arrest prior to intervention; pre-catheterization therapy with lytic agents, or with glycoprotein IIb/IIIa inhibitors, or with pacemakers; life expectancy <1 year; current participation in other investigations	1 month	Reperfusion (rate of ST-segment resolution >50 % at 60 minutes postprocedure or MBG 3 immediately postprocedure); magnitude of ST-segment resolution; improvement in TIMI flow; corrected TIMI frame count; MACE (death, recurrent MI, emergent CABG, TLR or TVR, stroke); rate of distal embolization; rate of required bailout techniques (rescue use of the aspiration catheter, distal protection, or glycoprotein IIb/IIIa inhibitors)
			C = 61.2 (12.9)					
IMPACT [35]	Cambridge, UK	56	I = 64.9 (11.2) C = 67.2 (11.6)	31 (55.3)	>18 and <90 years old, ability to give informed consent, STEMI (ST-segment elevation ≥2 mm in ≥2 contiguous chest leads or ≥1 mm in ≥2 contiguous limb leads) or new LBBB, chest pain for <12 hours, restoration of at least TIMI 1 flow after the wire crossed the occlusion	Cardiogenic shock; previous MI in the IRA territory; unfavourable anatomy (LMCA occlusion or distal vessel occlusion); severe asthma or bradycardia precluding use of adenosine; women of childbearing age; life expectancy <3 months	6 months	Index of microcirculatory resistence; MACE (all-cause death or MI)
INFUSE-AMI [36, 37]	37 sites in 6 countries	452	I = 61 (NR)	334 (73.9)	≥18 years old, STEMI with ≥1 mm of ST-segment elevation in ≥2 contiguous leads in V1 through V4 or new LBBB with anticipated symptom onset to device time of ≤5 hours	Prior MI, CABG or LAD stenting; contraindications to study medications, contrast or CMRI; creatinine clearance <30 mL/min per 1.73 m^2^ or dialysis; platelet count <100,000 or >700,000 cells/mm^3^; hemoglobin <10 g/dL; recent major bleeding; bleeding diathesis; current warfarin use; intracranial disease, stroke or TIA within 6 months or any neurological defect; cardiogenic shock; prior fibrinolysis or glycoprotein IIb/IIIa inhibitors for the present admission; any comorbid likely to interfere with protocol compliance or associated with <1 year survival	12 months	Infarct size measured as a percentage of LV mass at 30 days. MACE (death, recurrent MI, new-onset severe HF, re-hospitalization for HF, stroke, clinically driven TVR)
			C = 60 (NR)					
ITTI [38]	Kaohsiung City, Yun-Lin Branch, Taiwan	100	I = 60.4 (11.9)	86 (86.0)	≥18 years old, continuous chest pain ≥30 min, ST-segment elevation >0.1 mV in ≥2 contiguous leads on a 12-lead ECG	Cardiogenic shock (systolic BP > 80 mmHg or need for inotropic agent); history of bleeding tendency, major operation within 6 weeks; hepatic or renal insufficiency; contraindication to tirofiban use	6 months	Occurrence of MBG 3; complete ST-segment resolution; procedure time; occurrence of no-reflow; CK-MB peak and time to peak; TIMI flow and corrected TIMI frame count; MACE (death, recurrent MI, TLR, stroke)
			C = 56.5 (11.9)					
Kaltoft 2006 [39]	Aarhus, Denmark	215	I = 65 (11)	168 (78.1)	STEMI, symptoms lasting >30 min but <12 hours, and cumulative ST-segment elevation of ≥2 mV in ≥2 contiguous leads	LBBB; MI within the previous 30 days; fibrinolytic treatment; previous CABG; LCA stenosis; need for mechanical ventilation; severe HF treated with intra-aortic balloon pump	1 month	Myocardial salvage estimated by 99mTc-sestamibi SPECT; final infarct size; markers of effective reperfusion (TIMI flow, corrected TIMI frame count, ST-segment resolution immediately, 90 min and 6 hours after PCI); release of TnT; distal embolization visible at the end of PCI; total procedure time; MACE (death, recurrent MI, disabling stroke); LVEF after 30 days; technical success of the thrombectomy
			C = 63 (13)					
Liistro 2009 [40]	Arezzo, Italy	111	I = 64 (11)	86 (77.5)	STEMI with symptoms lasting >30 minutes and <12 hours, ST-segment elevation >0.1 mV in ≥2 leads on the ECG	Contraindication to the use of platelet glycoprotein IIb/IIIa inhibitors; rescue PCI after thrombolysis; previous MI; absence of optimal echocardiographic apical view; life expectancy <6 months; lack of informed consent	6 months	Rate of ST-segment resolution ≥70 %; TIMI 3 grade flow; corrected TIMI frame count; myocardial contrast echocardiography score index; absence of persistent ST-segment deviation; time course of wall-motion score index; LVEF; LV volume; death; recurrent MI; LV failure; new revascularization
			C = 65 (11)					
REMEDIA [41]	Rome, Italy	99	I = 61 (13)	83 (83.3)	<12 hours of onset of STEMI referred for primary or rescue PCI	No angiographic exclusion criteria were adopted	1 month	MBG ≥2; rate of ST-segment resolution ≥70 %; peak CK-MB; direct stenting rate; distal embolization rate (abrupt “cutoff” occlusion of a distal branch); composite of distal embolization, slow-flow (TIMI flow grade 2), no-reflow (TIMI flow grade 0 to 1); death; recurrent MI; stroke; TLR; any major adverse event
			C = 60 (13)					
Shehata 2014 [25]	Cairo, Egypt	100	I = 60.32 (9.2)	64 (64)	Diabetic patients suffering from acute STEMI, symptoms lasting >30 minutes and <12 hours before admission, and ST-segment elevation of >0.1 mV in ≥2 leads	Need for rescue PCI after thrombolysis; prior history of unstable angina or MI; prior PCI CABG; congenital heart disease or any myocardial disease apart from ischemia; limited life expectancy due to coexistent disease	8 months	In-stent restenosis (angiographic luminal diameter stenosis by >50 % in quantitative coronary angiography); MACE (death due to cardiac cause, nonfatal MI, TLR)
			C = 59.4 (7.4)					
Sim 2013 [42]	Gwangju, Republic of Korea	86	I = 63 (NR)	59 (71.1)	STEMI with onset of symptoms <12 hours, coronary artery lesions with visible thrombus, ability to undergo a complete CCT examination (Killip I and II) with the ability to perform a15-second breath-hold	Previous MI or CABG; cardiogenic shock; LMCA disease; severe valvular heart disease; unsuccessful PCI (post-PCI TIMI flow <2 or ≥50 % residual stenosis in IRA); rescue or facilitated PCI; contraindication to glycoprotein IIb/IIIa inhibitors	12 months	Infarct size at 2 months; markers of myocardial reperfusion (TIMI flow, MBG, ST-segment resolution rate at 90 min); LV function and volumes at 2 months; MACE (cardiac death, MI, TVR)
			C = 60(NR)					
TAPAS [43, 44]	Groningen, The Netherlands	1071	I = 63 (13)	755 (70.5)	STEMI, symptoms >30 minutes and <12 hours, and ST-segment elevation of ≥0.1 mV in ≥2 leads	Rescue PCI after thrombolysis; life expectancy <6 months; lack of informed consent	1 month	Rate of post-procedural MBG of 0; rate of TIMI flow grade of 3; complete resolution of ST-segment elevation; absence of persistent ST-segment deviation; TVR; recurrent MI; death
			C = 63 (13)					
TASTE [26, 27]	29 centers in Sweden, 1 center in Iceland and 1 in Denmark	7244	I = 66.5 (11.5)	5424 (74.9)	STEMI, chest pain for >30 minutes and <24 hours, ST-segment elevation in ≥2 contiguous leads (≥0.2 mV in lead V2 or V3 or ≥0.1 mV in other leads) or a presumably new LBBB, and a corresponding culprit-artery lesion on angiography	Need for emergency CABG; inability to provide oral informed consent; <18 years old; previously randomized in the study	12 months	MACE (all-cause mortality; rehospitalization for MI; stent thrombosis); TVR; TLR; complications of PCI, stroke or neurologic complications, HF and length of stay during index hospitalization
			C = 65.9 (11.7)					
TOTAL [6]	87 hospitals in 20 countries	10732	I = 61.0 (11.8)	7797 (72.6)	Symptoms of MI lasting for ≥30 min, definite ECG changes indicating STEMI, referred for PCI for presenting symptoms, randomized within 12 hours of symptoms onset and before diagnostic angiography, Informed consent	≤18 years old; prior CABG; life expectancy <6 months due to noncardiac condition; treatment with fibrinolytic therapy for qualifying index STEMI event	6 months	MACE (cardiovascular death, recurrent MI, cardiogenic shock, HF NYHA class IV); stroke
			C = 65.0 (11.9)					
TROFI [45, 46]	5 european centres	141	I = 61.1 (11.8)	102 (72.3)	≥18 years old, STEMI documented with ≥2 mm ST-segment elevation in ≥2 contiguous leads prior to PCI, presenting in the cath lab <12 hours after the onset of symptoms lasting ≥20 min and having an angiographically visible stenosis (>30 %) or TIMI ≤ II in a single de novo, native, previously unstented vessel	Pregnancy; known intolerance to aspirin, clopidogrel, heparin, stainless steel, limus drugs, contrast material; diameter stenosis <30 % in the target lesion; multi-vessel CAD; unprotected LMCA stenosis >30 %; distal vessel occlusion; severe tortuous, calcified or angulated anatomy that would result in sub-optimal imaging or excessive risk of complication from insertion of catheter; fibrinolysis prior to PCI; platelet <100,000 cells/μl; coagulopathy or active bleeding or chronic anticoagulation therapy; cardiogenic shock; significant comorbidities precluding follow-up as judged by investigators; major planned surgery requiring discontinuation of antiplatelets; proximal RCA stenosis (>30 %) if the IRA is mid or distal-RCA	12 months	Minimum flow area immediately after PCI assessed by OFDI; MACE (cardiac death, recurrent MI in the territory of IRA, clinically driven TVR)
			C = 60.9 (12.7)					
VAMPIRE [47]	23 hospitals in Japan	355	I = 63.2 (10.6)	281 (79.1)	≥21 years old, STEMI symptom >30 min but <24 hours, ST-segment elevation ≥2 mm in ≥2 contiguous leads or with a presumably new LBBB	Primary thrombolysis prior to randomization; cardiogenic shock; history of cardiac arrest; history of CABG; chronic renal failure (Cr >2.0 mg/dl) or hemodialysis; LMCA disease; target vessel <2.5 mm or >5 mm in diameter	8 months	Incidence of slow flow or no reflow during primary PCI (TIMI flow grade <3 not attributable to dissection, occlusive thrombus, or epicardial spasm); coronary flow and myocardial perfusion immediately after PCI (assessed by TIMI flow grade, corrected TIMI frame count and MBG); magnitude of ST-segment resolution, peak CK and CK-MB; angiographic in-stent late lumen loss; LV function; brain natriuretic peptide; MACE (death, recurrence MI, TLR)
			C = 63.5 (9.9)					
Yin 2011 [48]	Dalian, China	164	I = 63.1 (12.9)	120 (73.2)	STEMI patients who had PCI	Not reported	12 months	Thrombus score; periprocedural no-reflow; TIMI frame count; lumen diameter; stent length; 1-week post-procedural ejection fraction; post-procedural angina; recurrent MI; death
			C = 62.9 (9.5)					

*SD* standard deviation, *no.* number, *I* intervention group, *C* control group, *STEMI* ST-segment elevation myocardial infarction, *TIMI* thrombolysis in myocardial infarction, *LV* left ventricular, *MACE* major adverse cardiac events, *MI* myocardial infarction, *TVR* target vessel revascularization, *ECG* electrocardiogram, *PCI* percutaneous coronary intervention, *LMCA* left main coronary artery, *IRA* infarct-related artery, *CABG* coronary artery bypass grafting, *TLR* target lesion revascularization, *MBG* myocardial blush grade, *HF* heart failure, *LBBB* left bundle branch block, *NR* not reported, *LAD* left anterior descending, *CMRI* cardiac magnetic resonance imaging, *TIA* transient ischemic attack, *SPECT single-photon emission computed tomography, TnT* troponin T, *LVEF* left ventricular ejection fraction, *CK-MB* creatine kinase myocardial band, *CCT* cardiac computed tomography, *NYHA* New York Heart Association, *CAD* coronary artery disease, *OFDI* optical frequency domain imaging, *RCA* right coronary artery

 Twelve studies [25, 28–30, 34, 35, 38–44] used aspirin and clopidogrel as a preprocedure antithrombotic therapy; some of them [6, 25–30, 32–35, 38, 39, 41–47] also used intravenous heparin; seven of them had all patients were treated with abciximab [25, 31, 35, 39, 40, 41, 43, 44] and; one of them [42] also used nitroglycerin (Table 2).

**Table 2:** Study protocol used as preprocedure reported by the included studies

Author, year	Different regimens of anti-aggregation/anticoagulation used
ADMIT [28]	Oral aspirin 300 mg as a loading dose (or only 100 mg if the patient was on aspirin therapy) continued by 100 mg/day indefinitely, 600 mg clopidogrel loading dose continued by 75 mg/day for one year and IV 60 mg/ kg unfractionated heparin as loading dose to keep activating clotting time during procedure > 250 second.
Bulum 2012 [29]	300 mg of aspirin and 600 mg of clopidogrel and a weight-adjusted dose of unfractionated heparin; the usage of glycoprotein IIb/IIIa inhibitor (eptifibatide) was left to the discretion of the operator.
Chao 2008 [30]	Aspirin 300 mg and clopidogrel 300 mg were given as loading dose, with intravenous heparin 70– 100 U/kg to achieve activated clotting time (ACT) > 200 s prior to intervention.
De Luca 2006 [31]	Aspirin 300 mg orally and heparin 8000 IU intravenously before the procedure and abciximab as a 0.25 mg/kg bolus and 0.125 mg/kg/min intravenous infusion immediately before the revascularisation and continued for 12 hours.
EXPIRA [32, 33]	Aspirin 300 mg, intravenous heparin, abciximab at a standard dose, and clopidogrel 300 mg before the revascularization.
EXPORT [34]	The choice of medication during the procedure such as aspirin, heparin, clopidogrel, and glycoprotein IIb/IIIa inhibitors was also at the investigator’s discretion, and were administrated according to standard hospital procedure.
IMPACT [35]	Aspirin 300 mg and clopidogrel 600 mg preloading in the ambulance and anticoagulated with a heparin bolus (70–100 U/kg) after arterial sheath insertion to achieve an activated clotting time (ACT) >250 s. Adjunctive pharmacotherapy, including abciximab and bivalirudin, was given at the operator’s discretion.
INFUSE-AMI [36, 37]	Patients undergoing primary PCI received bivalirudin anticoagulation.
ITTI [38]	Aspirin (300 mg loading followed by 100 mg daily) and clopidogrel (300 mg loading followed by 75 mg daily) and unfractionated heparin 100 IU/kg.
Kaltoft 2006 [39]	Aspirin 300 mg orally or intravenously, clopidogrel 300 mg orally, and unfractionated heparin 10 000 IE intravenously. During the intervention, all patients were treated with abciximab.
Liistro 2009 [40]	Aspirin (a loading dose of 500 mg), heparin (70 IU/kg), and clopidogrel (a loading dose of 600 mg). All patients also received the glycoprotein IIb/IIIa inhibitor abciximab with an intravenous procedural bolus of 0.25 mg/kg followed by a continuous intravenous infusion of 0.125 μg/kg/min for 12 hours and postprocedural infusion without heparin.
REMEDIA [41]	Heparin (initial weight-adjusted IV bolus then further boluses administered with the aim of obtaining an activated clotting time of 250 to 300 s in patients treated with abciximab and > 300 s in the remaining subjects) and with double antiplatelet therapy with aspirin and clopidogrel (loading dose of 300 mg followed by 75 mg/day) for at least four weeks. Unless contraindicated, abciximab (0.25 mg/kg bolus plus infusion of 0.125 μg/kg/min for 12 h) was intravenously administered in all patients undergoing primary PCI, whereas in those with failed thrombolysis, abciximab use was left to the operator’s discretion.
Shehata 2014 [25]	Aspirin (a loading dose of 500 mg), heparin (70 IU/kg), and clopidogrel (a loading dose of 600 mg). All patients also received the glycoprotein IIb/IIIa inhibitor abciximab with an intravenous procedural bolus of 0.25 mg/kg followed by a continuous intravenous infusion of 0.125 g/kg/min for 12 hours and postprocedural infusion without heparin.
Sim 2013 [42]	Aspirin 300 mg, clopidogrel 600 mg, intravenous unfractionated heparin and nitroglycerin. Oral atenolol 50–100 mg was given to optimize heart rate ≤ 65 beats per minute prior to CT scan, unless contraindicated.
TAPAS [43, 44]	Aspirin (a loading dose of 500 mg), heparin (5000 IU), and clopidogrel (a loading dose of 600 mg). Patients also received the glycoprotein IIb/IIIa inhibitor abciximab, with the dose based on body weight, unless contra-indicated, and additional heparin, with the dose based on the activated clotting time.
TASTE [26, 27]	Patients received the following procedure-related medication: bivalirudin, clopidogrel or ticlopidine, acetylsalicylic acid, ticagrelor, prasugrel, heparin, low-molecular-weight heparin, and glycoprotein IIb/IIIa blocker. The use of platelet inhibitors or anticoagulants was left to the discretion of the treating physician.
TOTAL [6]	Unfractionated heparin; bivalirudin; enoxaparin and; glycoprotein IIb/IIa inhibitor.
TROFI [45, 46]	Heparin in ambulance.
VAMPIRE [47]	Aspirin and intravenous heparin boluses were administered during the procedure to maintain an activated clotting time ≥ 300 s.
Yin 2011 [48]	Aspirin 300 mg and clopidogrel 300 mg prior to angiography.

IV: intravenous

The choice of medication during the procedure such as aspirin, heparin, clopidogrel, and glycoprotein IIb/IIIa inhibitors was at the investigator’s discretion in one of the included studies [34]. The patients in one further trial [26, 27] received the following procedure-related medication: bivalirudin, clopidogrel or ticlopidine, acetylsalicylic acid, ticagrelor, prasugrel, heparin, low-molecular-weight heparin, and glycoprotein IIb/IIIa blocker, while in other one [6] patients received unfractionated heparin; bivalirudin; enoxaparin and; glycoprotein IIb/IIa inhibitor (Table 2). Patients in TROFI trial [45, 46] received only heparin in ambulance and, in VAMPIRE trial [47] aspirin and intravenous heparin boluses were administered during the procedure to maintain an activated clotting time ≥ 300 s.

### Risk of bias assessment

**Fig. 2:**
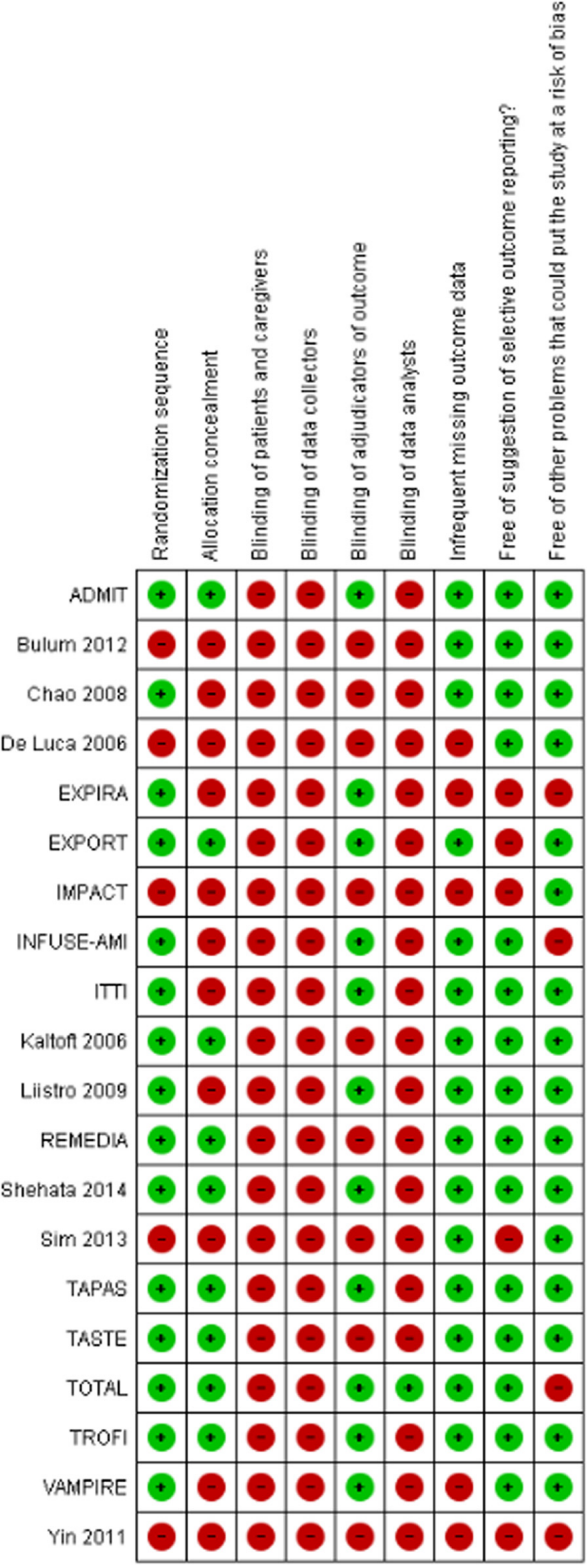
Risk of bias assessment

A possibly important limitation with respect to risk of bias was lack of blinding for caregivers. A number of studies, including the larger ones, blinded the adjudicators of outcome. Follow-up was largely satisfactory: 14 trials lost less than 10 % of patients to follow-up (Table 3 and Fig. 2).

**Table 3:** Risk of bias assessment

Author, year	Randomization sequence adequately generated?	Allocation adequately concealed?	Blinding of patients and caregivers?	Blinding of data collectors?	Blinding of adjudicators of outcome?	Blinding of data analysts?	Infrequent missing outcome data?^a^	Free of suggestion of selective outcome reporting?﻿	Free of other problems that could put it at a risk of bias?
ADMIT (28)	Yes	Yes	No	Probably no	Probably yes	Probably no	Yes	Yes	Yes
Bulum 2012 (29)	Probably no	Probably no	No	No	No	No	Yes	Yes	Yes
Chao 2008 (30)	Probably yes	Probably no	No	No	No	No	Yes	Probably yes	Yes
De Luca 2006 (31)	Probably no	Probably no	No	Probably no	Probably no	Probably no	No	Yes	Yes
EXPIRA (32, 33)	Probably yes	Probably no	No	No	Yes	No	Probably yes	Probably yes	Probably yes
EXPORT (34)	Yes	Yes	No	No	Yes	No	Yes	Probably no	Probably yes
IMPACT (35)	Probably no	Probably no	No	Probably no	Probably no	Probably no	No	No	Yes
INFUSE-AMI (36, 37)	Yes	Probably no	No	Probably no	Yes	Probably no	Yes	Yes	No
ITTI (38)	Yes	Probably no	No	Probably no	Probably yes	Probably no	Yes	Yes	Yes
Kaltoft 2006 (39)	Yes	Yes	No	Probably no	Probably no	Probably no	Yes	Yes	Yes
Liistro 2009 (40)	Yes	Probably no	No	No	Probably yes	No	Probably yes	Yes	Yes
REMEDIA (41)	Yes	Probably yes	No	No	No	No	Probably yes	Yes	Probably yes
Shehata 2014 (25)	Yes	Yes	No	Probably no	Yes	Probably no	Yes	Yes	Yes
Sim 2013 (42)	Probably no	Probably no	No	No	No	No	Yes	Probably no	Yes
TAPAS (43, 44)	Yes	Probably yes	No	No	Yes	No	Yes	Yes	Yes
TASTE (26, 27)	Yes	Yes	No	No	No	Probably no	Yes	Yes	Yes
TOTAL (6)	Yes	Yes	No	Probably no	Yes	Probably yes	Yes	Yes	Probably no
TROFI (45, 46)	Yes	Yes	No	No	Yes	Probably no	Yes	Yes	Yes
VAMPIRE (47)	Probably yes	Probably no	No	No	Yes	No	No	Yes	Probably yes
Yin 2011 (48)	No	No	No	No	No	No	No	No	Probably no

^a^Defined as less than 10 % loss to outcome data or difference between groups less than 5 % and those excluded are not likely to have made a material difference in the effect observed

All answers as: yes (low risk of bias), probably yes, probably no, no (high risk of bias)

### Outcomes

Appendix Table 2 presents the mortality data by individual study and Appendix Table 3 presents individual study outcome data for recurrent MI, stroke, and bleeding.

#### Overall mortality

**Fig. 3:**
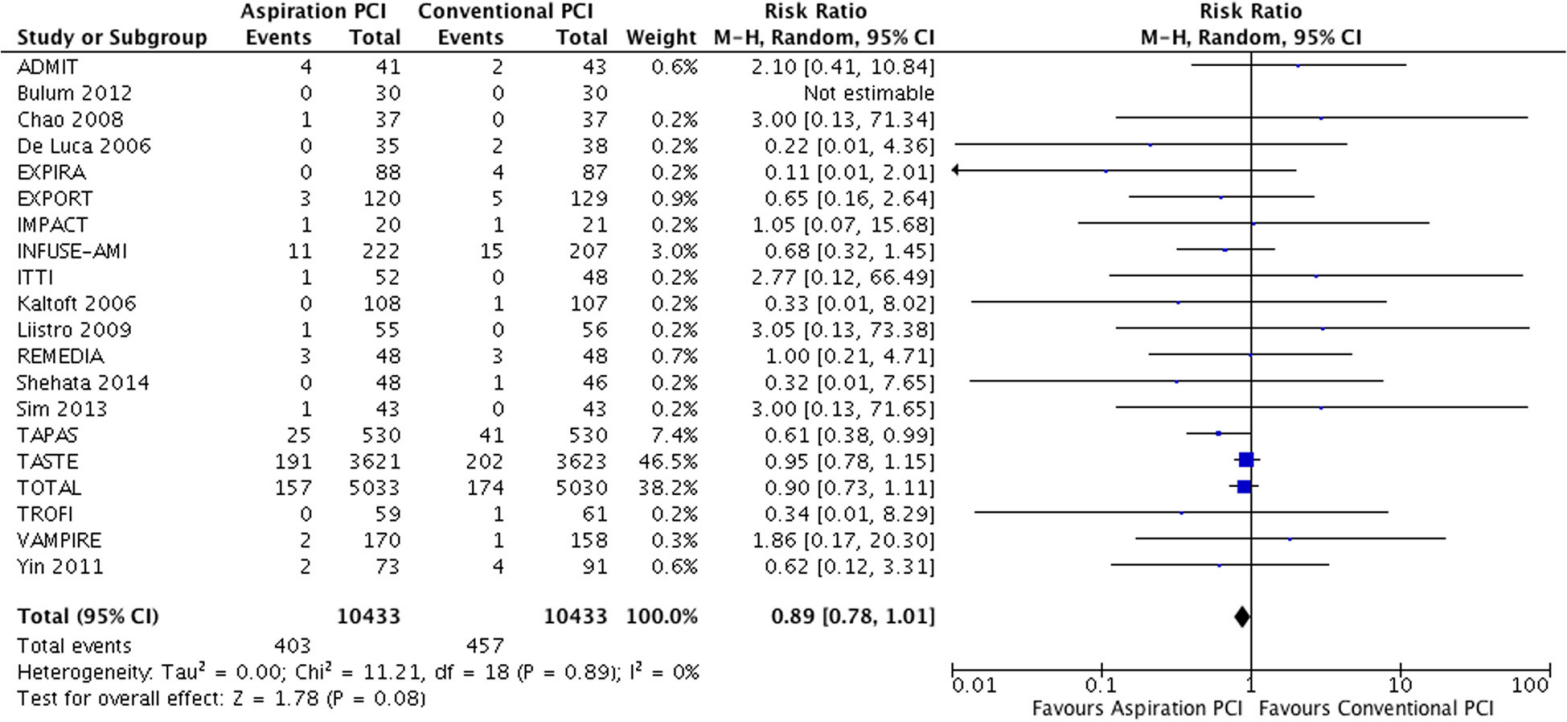
Meta-analysis comparing aspiration PCI versus conventional PCI on overall mortality

In 20 trials [6, 25–48] that addressed overall mortality, 457 of 10,433 (4.4 %) patients died in the control arm compared to 403 of 10,433 (3.9 %) in the aspiration PCI arm (relative risk (RR) 0.89, 95 % CI 0.78 to 1.01; I^2^ = 0 %; risk difference (RD) 4/1,000 over 6 months; moderate certainty) (Fig. 3). Certainty in evidence was rated down to moderate because of imprecision and unblinding of caregivers in all included studies (Table 4).

**Table 4:** *GRADE evidence profile:* Aspiration thrombectomy (AT) prior to PCI in patients with STEMI

Quality assessment						Summary of findings					Certainty in estimates
						Study event rates		Relative risk (95 % CI)	Anticipated absolute effects over6 months		OR Quality of evidence
No of participants(studies) Range follow-up time	Risk of bias	Inconsistency	Indirectness	Imprecision	Publication bias	Without AT	With AT		Without AT	With AT	
Overall mortality (Includes cardiovascular (CV) mortality for studies only reporting CV mortality)											
20866 (20) 6–12 mo	No serious limitations^1^	No serious limitations	No serious limitations^2^	Serious imprecision^1,3^	Undetected	457/ 10433	403/ 10433	0.89 (0.78-1.01)	35 per 1000^4^	4 fewer per 1000 (8 fewer to 0 more)	⊕⊕⊕⊕O MODERATE, due to imprecision
Recurrent myocardial infarction											
20662 (17) 6–12 mo	No serious limitations ^1^	No serious limitations	No serious limitations	Serious imprecision^1,5^	Undetected	246/ 10331 (2.3 %)	229/10331(2.2 %)	0.94 (0.79-1.12)	18 per 1000^4^	1 fewer per 1000 (4 fewer to 2 more)	⊕⊕⊕⊕O MODERATE, due to imprecision
Stroke											
18348 (8) 6–12 mo	No serious limitations ^1^	No serious limitations	No serious limitations	Serious imprecision^1,6^	Undetected	48/ 9163 (0.5 %)	77/9185 (0.8 %)	1.56 (1.09-2.24)	5 per 1000^4^	3 more per 1000 (0 more to 6 more)	⊕⊕⊕⊕O MODERATE, due to imprecision
Major bleeding											
11655 (4) 6–12 mo	No serious limitations ^1^	No serious limitations	No serious limitations	Serious imprecision^1,5^	Undetected	99/5823 (1.7 %)	101/5832 (1.7 %)	1.02 (0.78-1.35)	15 per 1000^4^	0 more per 1000 (3 fewer to 5 more)	⊕⊕⊕⊕O MODERATE, due to imprecision

^1^No studies were blinded to patient or caregiver. Some studies (minority of subjects enrolled) did not indicate blinded adjudication. While not specifically rating down for risk of bias, these additional concerns plus borderline clinically important imprecision led to downgrading of certainty in estimates for all outcomes

^2^Some studies only report cardiovascular and not all cause mortality. However cardiovascular mortality constituted significant proportion of overall mortality in studies reporting both types of mortality. Therefore we opted against rating down for indirectness

^3^95% CI for absolute effects include clinically important benefit and no benefit

^4^Baseline risk estimates for mortality, recurrent MI, stroke, and major bleeds come from control arm of TOTAL study (largest and most recent randomized trial)

^5^95% CI for absolute effects include benefit and harm

^6^95% CI for absolute effects include clinically important harm and no harm

#### Recurrent myocardial infarction

**Fig. 4:**
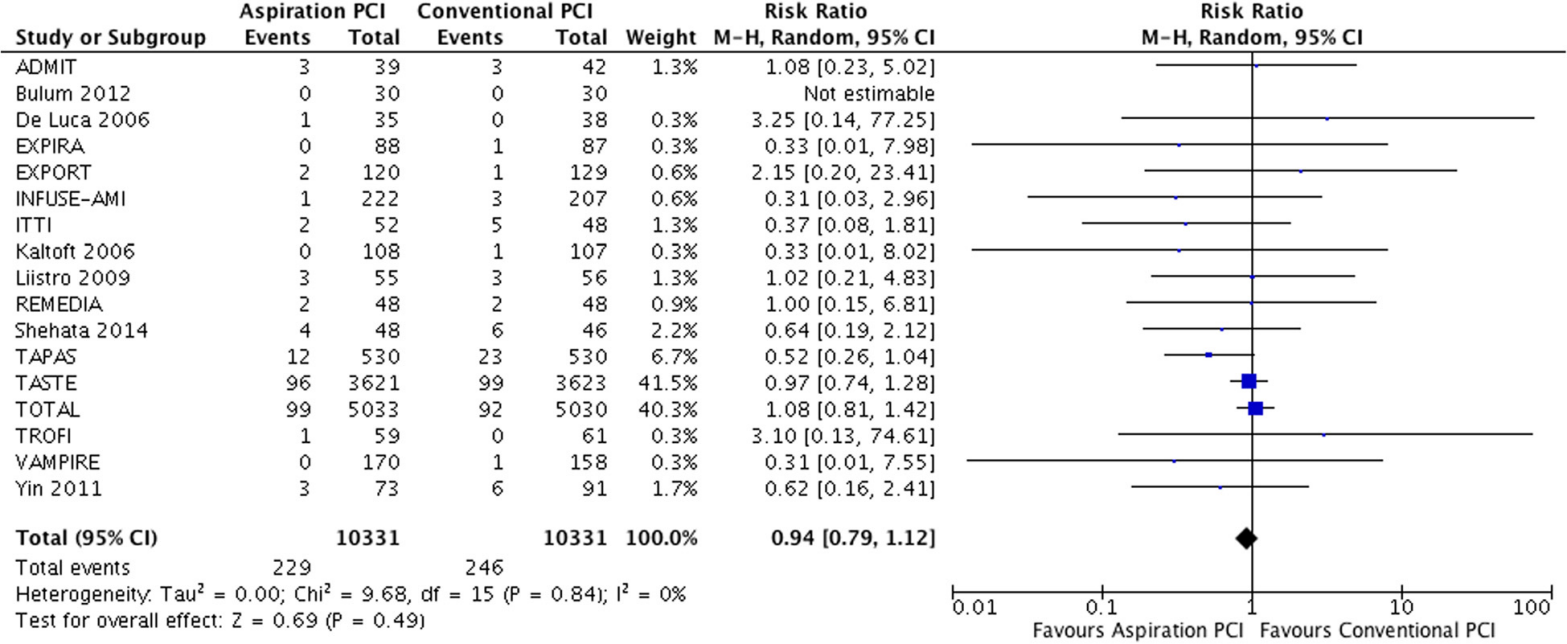
Meta-analysis comparing aspiration PCI versus conventional PCI on recurrent myocardial infarction

In 17 trials [6, 25–29, 31–34, 36–41, 43–48], 246 of 10,331 (2.4 %) patients suffered a recurrent MI in the control arm compared to 229 of 10,331 (2.2 %) in the aspiration PCI arm (RR 0.94, 95 % CI 0.79 to 1.12; I^2^ = 0 %; RD 1/1,000 over 6 months; moderate certainty) (Fig. 4). Certainty in evidence was rated down to moderate because of imprecision, lack of blinding of caregivers in all included studies and inadequate or unreported blinding of outcome adjudicators in some studies [26, 27, 29, 31, 39, 41, 48] (Table 4).

#### Stroke

**Fig. 5:**
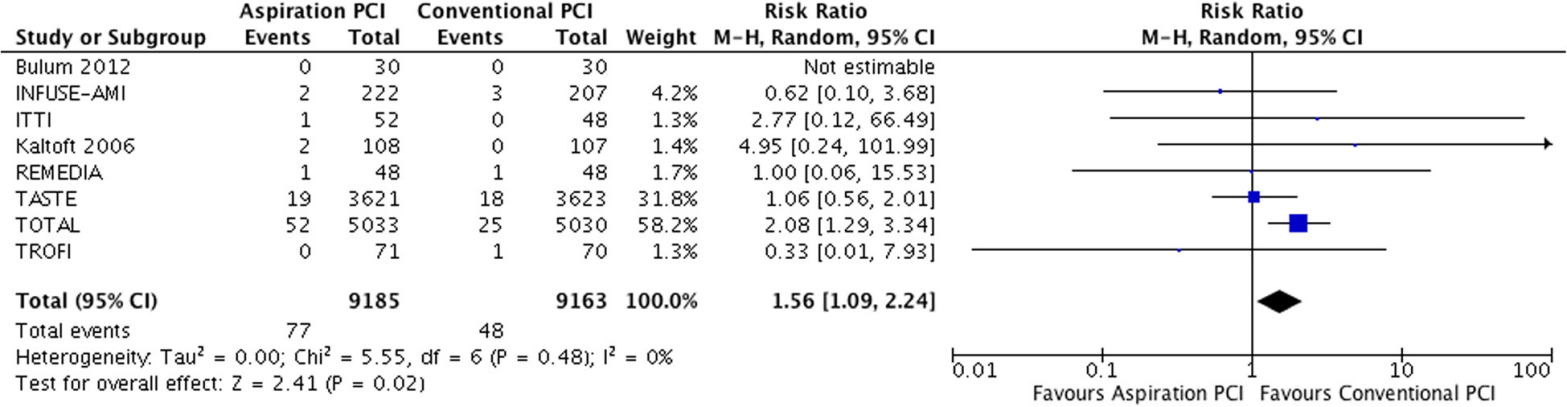
Meta-analysis comparing aspiration PCI versus conventional PCI on stroke.

In 8 trials [6, 26, 27, 29, 36–39, 41, 45, 46], 77 of 9,185 (0.8 %) patients that underwent aspiration PCI use had a stroke compared to 48 of 9,162 (0.5 %) in the PCI alone (RR 1.56, 1.09 to 2.24; I^2^ = 0 %; RD 3/1,000 over 6 months; moderate certainty) (Fig. 5). Certainty in evidence was rated down to moderate because of imprecision, lack of blinding of caregivers in all included studies and inadequate or unreported blinding of outcome adjudicators in some studies [26, 27, 29, 39, 41] (Table 4). We intended to evaluate non-fatal stroke, but data was not available in sufficient number of studies to provide a useful comparison.

#### Major bleeding

**Fig. 6:**
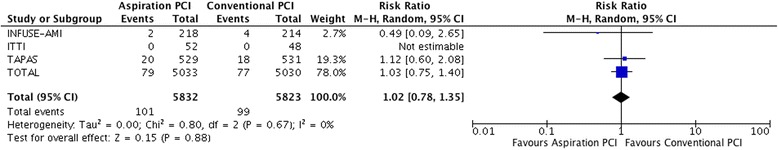
Meta-analysis comparing aspiration PCI versus conventional PCI on major bleeding

In 4 trials [6, 36–38, 43, 44], 99 of 5823 (1.7 %) patients presented major bleeding in the control arm compared to 101 of 5,832 (1.7 %) in the aspiration PCI arm (RR 1.02, 0.78 to 1.35; I^2^ = 0 %; RD 0/1,000 over 6 months; moderate certainty) (Fig. 6). Certainty in evidence was rated down to moderate because of imprecision and lack of blinding of caregivers in all included studies (Table 4).

More than 10 studies addressed overall mortality and recurrent MI; for both, funnel plots did not suggest publication bias (Appendix: Figures 1 and 2).

#### Meta-Regression analysis

**Fig. 7:**
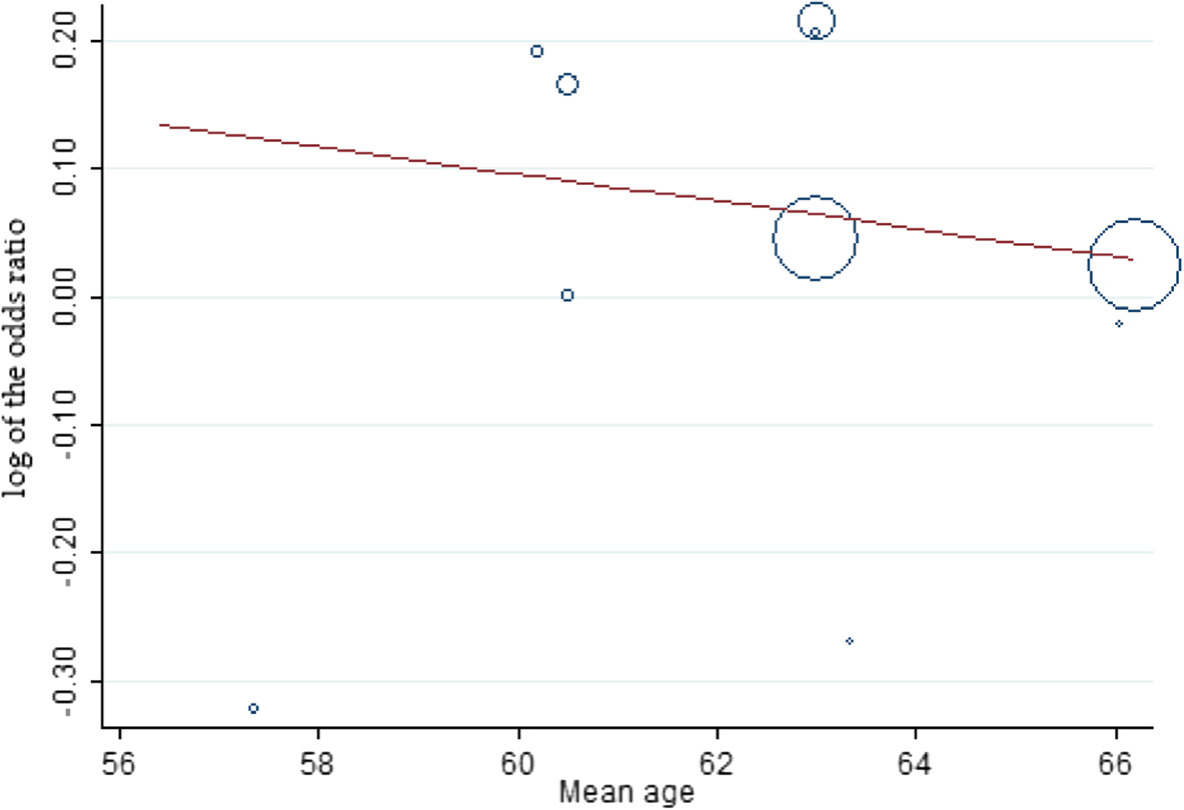
Meta-regression of mortality rates by mean age. Each circle represents a study highlighted by its weight in the analysis. The relationship between mortality and mean age in both groups was not significant (slope: -0.011; 95 % confidence interval: -.0980 to .0765; P = 0.784)

**Fig. 8:**
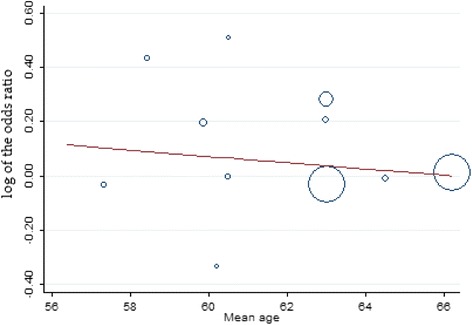
Meta-regression of recurrent myocardial infarction rates by mean age. Each circle represents a study highlighted by its weight in the analysis. The relationship between recurrent myocardial infarction and mean age in both groups was not significant (slope: -0.011; 95 % confidence interval: -.1175 to .0944; *P* = 0.811)

Data from studies assessed in a meta-regression showed that the relationship between mortality rates decreased with increasing mean age; however was not significant (slope: −0.011; 95 % confidence interval: −.0980 to .0765; P = 0.784; Fig. 7). Similarly, the relationship between recurrent myocardial infarction rates decreased with increasing mean age; however was not significant (slope: −0.011; 95 % confidence interval: −.1175 to .0944; P = 0.811; Fig. 8).

## Discussion

### Main findings

Based on pooled data from 20 randomized trials with more than 20,000 patients, we found moderate quality evidence for a non-statistically significant reduction in overall mortality (4 fewer deaths/1000 treated over 6 months) (Table 4) and a small potential increase in stroke (3 additional strokes/1000 treated over 6 months) (Table 4) in patients treated with thrombectomy. Moderate quality evidence suggests no impact of thrombectomy on either recurrent MI or major bleeding (Table 4).

A number of factors decreased our certainty in the estimates for overall mortality. In particular, the confidence interval included both no reduction in deaths and a mortality reduction that although small (8 fewer deaths in 1,000 over six months), many would consider important. Similarly with stroke: the confidence interval includes no increase in stroke and an increase of 6 more strokes in 1,000 patients over 6 months with thrombectomy, which many would consider an important risk. Other issues decreasing confidence in our estimates included potential risk of bias imposed by lack of blinding of patients and health care providers in all studies, and lack of blinding of outcome adjudicators in some studies.

The meta-regression analyses showed that both mortality and recurrent myocardial infarction rates decreased with increasing mean age. However, there was a non-significant difference between these two variables and the mean age of participants in both studied groups. A study [49] evaluated through a meta-regression whether there is an association between age, gender, diabetes mellitus, previous myocardial infarction and ejection fraction, and the choice of revascularization, focusing on death, myocardial infarction, repeat revascularization and stroke. The authors found that the reduction in stroke was significantly higher in females, and that women and patients with diabetes mellitus were at increased risk of subsequent revascularization after PCI [49].

### Strengths and limitations

Strengths of our review include a comprehensive search; assessment of eligibility, risk of bias, and data abstraction independently and in duplicate; use of the GRADE approach in rating the quality of evidence for each outcome; and focus on absolute as well as relative effects of the intervention on patient-important outcomes. In this case, the small and more or less equivalent number of possible deaths prevented and strokes caused by thrombectomy, and the uncertainty consequent on the imprecision and risk of bias issues, are crucial in considering patient management (Table 4).

Potential limitations are related to the available data. Trials often suffered from incomplete outcome reporting, and lack of blinding consequent on the nature of the intervention, but for some studies also avoidable lack of blinding (outcome adjudication).

### Relation to prior work

Recently published results from another meta-analysis [50] as well as data from a limited meta-analysis conducted as part of an evaluation of the outcome of stroke in the TOTAL study [12] are in general consistent with our findings. Results from all three analyses are in general consistent with our findings. Our systematic review and meta-analysis nevertheless adds important information as a result of our comprehensive assessment of risk of bias issues, our use of a complete case analysis that avoids assumptions regarding patients lost to follow-up, our use of the GRADE approach to rate quality of evidence, and our focus on absolute effects of thrombectomy required for optimal decision-making.

Furthermore, another review compared the effects of thrombectomy as an adjunct to PCI in the management of acute myocardial infarction in 20,853 patients [51]. The authors concluded that mortality; reinfarction and; stent thrombosis rates did not differ significantly between patients treated with or without AT; but stroke rates were increased with AT [51].

### Implications

The possible magnitude of benefit with respect to mortality and magnitude of harm with respect to stroke are small – some might say very small – and similar both with respect to magnitude and likelihood that the effects are real. With respect to mortality, the most likely mechanism of benefit would be a reduction in recurrent MI; the data, however, provide no support for an impact of thrombectomy on MI.

Similarly the mechanism of an increase in stroke is not immediately apparent. In a recent analysis of data from the TOTAL study, thrombectomy was associated with a small increase in procedure time as well as increased use of larger catheters (99.2 % vs. 97.5 % > 5 French) [12]. One could postulate this could lead to an increase in embolization of aortic atherosclerotic plaque leading to increased early ischemic events. More frequent development of subsequent atrial fibrillation would constitute another possible mechanism; no study reported this outcome.

Initial enthusiasm for thrombectomy was motivated by evidence of improvement in markers of myocardial tissue reperfusion. Our findings emphasize the need for caution with respect to surrogates, and the desirability of focus on outcomes important to patients. While it is not routinely justified there may be individual cases in which an operator may feel the potential benefit of the procedure outweighs potential risks.

The absolute effects of thrombectomy prior to primary PCI are very small and still associated with uncertainty. Given the best estimates of effect and associated quality of evidence, fully informed risk adverse patients - and particularly those who are highly stroke risk averse - would likely decline thrombectomy. Patients who place high value on an uncertain mortality reduction and have limited concern regarding a possible stroke increase would be more likely to choose to undergo the procedure. Given current concerns regarding overtreatment and efficient use of health care resources, a policy decision to not use thrombectomy in a particular catheterization laboratory is defensible.

## Conclusions

Moderate certainty evidence suggests aspiration thrombectomy is associated with a possible small decrease in mortality (4 less deaths/1000 over 6 months) and a small increase in stroke (3 more strokes/1000 over 6 months). Because absolute effects are very small and closely balanced, thrombectomy prior to primary PCI should not be used as a routine strategy.

## Abbreviations

AT: aspiration thrombectomy; CV: cardiovascular; CENTRAL: cochrane controlled trials register; CIs: confidential intervals; GRADE: grading of recommendations assessment, development and evaluation; MeSH: medical subject headings; MI: myocardial infarction; PRISMA: preferred reporting items for systematic reviews and meta-analyses statement; PCI: primary percutaneous intervention; RCTs: randomized controlled trials; RevMan: review manager; RRs: risk ratios; STEMI: ST-segment elevation MI; TOTAL: Trial of Routine Aspiration Thrombectomy with PCI versus PCI Alone in Patients with STEMI

## Figures and Tables

**Fig. 9:**
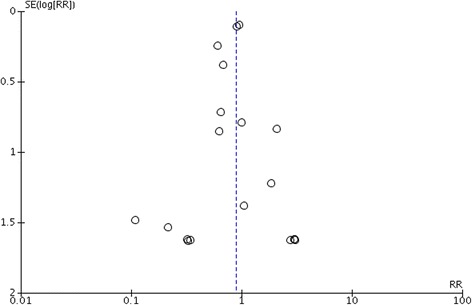
ᅟ

**Fig. 10:**
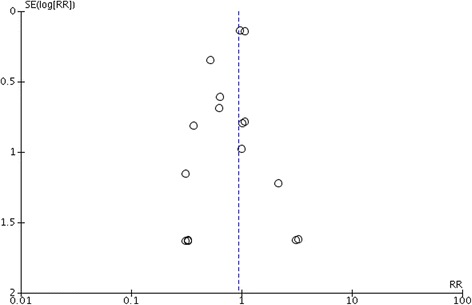
ᅟ

## Appendix

**Table 5:** Search strategy

Ovid MEDLINE(R) 1946 to present with daily update		
Ovid MEDLINE(R) in-process & other non-indexed citations June 24, 2015		
1.	myocardial infarction.ti,ab	194029
2	*Infarction/	4551
3	Myocardial Infarction/	145002
4	or/1-3	201604
5	thrombus aspiration.ti,ab.	400
6	thromboaspiration.ti,ab.	125
7	(aspiration adj5 mechanical).ti,ab.	214
8	Thrombectomy.ti,ab.	4995
9	(aspiration and catheter*).ti,ab.	2140
10	thrombosuction.ti,ab.	34
11	*Thrombectomy/	2028
12	or/5-11	7869
13	randomized controlled trial.pt.	398533
14	controlled clinical trial.pt.	89780
15	randomized.ab.	324620
16	placebo.ab.	163833
17	drug therapy.fs.	1786167
18	randomly.ab.	233298
19	trial.ab.	336144
20	groups.ab.	1465972
21	or/13-20	3564150
22	and/4,12,21	349
23	exp animals/ not humans.sh.	4063058
24	22 not 23	346
Embase 1974 to 2015 June 24		
1.	Myocardial Infarction.ti,ab.	138908
2	heart infarction/ or acute heart infarction/ or infarction/ or ST segment elevation myocardial infarction/	298819
3	myocardial disease/	4499
4	or/1-3	335897
5	thrombus aspiration.ti,ab.	899
6	thromboaspiration.ti,ab.	227
7	(aspiration adj5 mechanical).ti,ab.	328
8	Thrombectomy.ti,ab.	7683
9	(aspiration and catheter*).ti,ab.	3379
10	thrombosuction.ti,ab.	59
11	*Thrombectomy/	1973
12	or/5-11	11913
13	random$.tw.	995701
14	factorial$.tw.	25787
15	(crossover$ or cross-over$).tw.	76738
16	placebo$.tw.	221322
17	(doubl$ adj blind$).tw.	158296
18	(singl$ adj blind$).tw.	16231
19	assign$.tw.	266556
20	allocat$.tw.	95221
21	volunteer$.tw.	195251
22	Crossover Procedure.sh.	43314
23	Double-blind Procedure.sh.	123817
24	Randomized Controlled Trial.sh.	377450
25	Single-blind Procedure.sh.	20454
26	or/13-25	1582267
27	animals/ not humans/	1258280
28	and/4,12,26	454
29	28 not 27	454
CENTRAL Issue 5 of 12, May 2015		
#1	myocardial infarction:ti,ab,kw (Word variations have been searched)	17426
#2	MeSH descriptor: [Infarction] explode all trees	18
#3	MeSH descriptor: [Myocardial Infarction] explode all trees	8885
#4	#1 or #2 or #3	17525
#5	thrombus aspiration:ti,ab,kw (Word variations have been searched)	151
#6	thromboaspiration:ti,ab,kw (Word variations have been searched)	10
#7	aspiration mechanical:ti,ab,kw (Word variations have been searched)	251
#8	thrombectomy:ti,ab,kw (Word variations have been searched)	336
#9	aspiration catheter*:ti,ab,kw (Word variations have been searched)	293
#10	thrombosuction:ti,ab,kw (Word variations have been searched)	4
#11	MeSH descriptor: [Thrombectomy] explode all trees	144
#12	#5 or #6 or #7 or #8 or #9 or #10 or #11	860
#13	#4 and #12	216
	In Trials	195

**Table 6:** Mortality data

Acronym (author, year)	No. included in analysis (intervention/ control)	Follow-up time (month)*	Cardiac-specific mortality (intervention/control)	Overall mortality (intervention/control)
ADMIT [28]	41/43	6		4/41; 2/43
	47/47	1		3/47; 1/47
Bulum 2012 [29]	30/30	6		0/30; 0/30
Chao 2008 [30]	37/37	6	NA	1/37; 0/37
De Luca 2006 [31]	35/38	6		0/35; 2/38
EXPIRA[32, 33]	88/87	24	0/88; 6/87	0/88; 6/87
	88/87	9	0/88; 4/87	0/88; 4/87
EXPORT [34]	120/129	1	3/120; 5/129	3/120; 5/129
IMPACT[35]	20/21	6	1/20; 1/ 21	1/20; 1/ 21
INFUSE AMI [36, 37]	222/207	12	NA	11/222; 15/207
	218/214	1		0/218; 1/214
ITTI [38]	52/48	6		1/52; 0/48
Kaltoft 2006 [39]	108/107	1	NA	0/108 ; 1/107
Liistro 2009 [40]	55/56	6	1/55; 0/56	1/55; 0/56
REMEDIA[41]	48/48	1	NA	3/48; 3/48
Shehata 2014 [25]	48/46	8	0/48; 1/46	0/48; 1/46
Sim 2013 [42]	43/43	12	NA	1/43; 0/43
TAPAS [43, 44]	530/530	12	19/530; 36/530	25/530; 41/530
	529/531	1	NA	11/529; 21/531
TASTE [26, 27]	3621/3623	12		295/3621; 316/3623
	3621/3623	1-12		191/3621; 202/3623
TOTAL [6]	5033/5030	6	157/5033; 174/5030	157/5033; 174/5030
TROFI [45, 46]	59/61	12	0/59; 1/61	0/59; 1/61
VAMPIRE [47]	170/158	8		2/170; 1/158
Yin 2011 [48]	73/91	12	NA	2/73; 4/91

*Preference for 6-month mortality, then any defined period closest to 6 months, however abstract in-hospital mortality if that is the only one available was excluded from review

**Table 7:** Outcome data per study

Author, year	No. included in analysis (intervention/ control)	Follow-up time (Month)	No. (%) of major bleeding (intervention/ control)	No. (%) of non-fatal stroke (intervention/ control)	No. (%) of recurrent myocardial infarction (intervention/ control)
ADMIT [28]	39/42	6			3(7.7)/3(7)
	42/46	1			2(4.7)/0
	49/51	0			1(2)/0
Bulum 2012 [29]	30/30	6		0/0	0/0
Chao 2008 [30]	37/37				
De Luca 2006 [31]	35/38	6			1/0
EXPIRA [32, 33]	88/87	24			0/1(1.14)
EXPORT [34]	120/129	1			2(0.016)/1(0.77)
IMPACT [35]	20/21	6			
INFUSE AMI [36, 37]	222/207	12	NA	2(0.9)/3(1.4)	1(0.45)/3(1.4)
	218/214	1	2(0.9)/4(1.86)	0/1(0.46)	1(0.45)/2(0.93)
ITTI [38]	52/48	6	0/0	1(1.92)/0(0)	2(3.84)/5(10.41)
Kaltoft 2006 [39]	108/107	1		2(1.85)/0(0)	0/1(0.93)
Liistro 2009 [40]	55/56				3(5.4)/3(5.3)
REMEDIA [41]	48/48	1		1(2)/1(2)	2(4)/2(4)
Shehata 2014 [25]	48/46	8			4(8)/6(13)
Sim 2013 [42]	43/43	12			
TAPAS [43, 44]	529/531	1	20(3.78)/18(3)		4(0.75)/10(1.88)
	530/530	12			12(2.26)/23(4.3)
TASTE [26, 27]	3621/3623	12		19(0.52)/18(0.4)*	96(2.7)/99(2.7)
	3621/3623	1			19(0.52)/31(0.85)
TOTAL [6]	5033/5030	6	79(1.5)/77(1.5)	52(1)/25(0.5)	99(2)/92(1.8)
	5033/5030	1		33(0.65)/16(0.32)	
TROFI [45, 46]	59/61	12	NA	NA	1(1.7)/0
	71/70	0	NA	0/1(1.4)	0/0
VAMPIRE [47]	170/158	8			0/1(0.6)
	178/171	0			0/1(0.6)
Yin 2011 [48]	73/91	12			3(4)/6(6.6)
