# Erratum to: Aspiration thrombectomy prior to percutaneous coronary intervention in ST-elevation myocardial infarction: a systematic review and meta-analysis

**DOI:** 10.1186/s12872-016-0332-1

**Published:** 2016-09-20

**Authors:** Regina El Dib, Frederick Alan Spencer, Erica Aranha Suzumura, Huda Gomaa, Joey Kwong, Gordon Henry Guyatt, Per Olav Vandvik

**Affiliations:** 1 Department of Anaesthesiology, Botucatu Medical School, Unesp – Univ Estadual Paulista, São Paulo, Brazil; 2 McMaster Institute of Urology, McMaster University, Hamilton, Ontario Canada; 3 Division of Cardiology, Department of Medicine, McMaster University, St. Joseph’s Healthcare - 50 Charlton Avenue East, Hamilton, Ontario Canada; 4 Research Institute - Hospital do Coração (HCor), São Paulo, Brazil; 5 Department of Pharmacy, Tanta Chest Hospital, Tanta, Egypt; 6 Division of Cardiology and Heart Education And Research Training (HEART) Centre, Department of Medicine and Therapeutics, Prince of Wales Hospital, and Institute of Vascular Medicine, The Chinese University of Hong Kong, Shatin, Hong Kong; 7 Department of Clinical Epidemiology and Biostatistics, McMaster University, Hamilton, Ontario Canada; 8 Department of Medicine, McMaster University, Hamilton, Ontario Canada; 9 Department of Medicine, Innlandet Hospital Trust-Division Gjøvik, Oppland, Norway; 10 Institute for Health and Society, Faculty of Medicine, University of Oslo, Oslo, Norway

## Erratum

Unfortunately, after publication of this article [1] it was noticed that the name of Huda Gomaa was misspelled. The corrected author list can be seen above and the original article has been updated to reflect this.
